# A nine-gene signature related to tumor microenvironment predicts overall survival with ovarian cancer

**DOI:** 10.18632/aging.102914

**Published:** 2020-03-24

**Authors:** Qi Ding, Shanshan Dong, Ranran Wang, Keqiang Zhang, Hui Wang, Xiao Zhou, Jing Wang, Kee Wong, Ying Long, Shuai Zhu, Weigang Wang, Huayi Ren, Yong Zeng

**Affiliations:** 1 Translational Medicine Center, The Affiliated Cancer Hospital of Xiangya School of Medicine, Central South University/Hunan Cancer Hospital, Changsha, China; 2 Engineering Technology Research Center for Diagnosis-Treatment and Application of Tumor Liquid Biopsy, Changsha, China; 3 Key Laboratory of Radiation Oncology, Department of Radiation Oncology, Hunan Cancer Hospital and The Affiliated Cancer Hospital of Xiangya School of Medicine, Central South University, Changsha, China; 4 The Fifth Department of Gynecological Oncology, The Affiliated Cancer Hospital of Xiangya School of Medicine, Central South University/Hunan Cancer Hospital, Changsha, China

**Keywords:** ovarian cancer, prognosis, tumor microenvironment, risk score, LASSO

## Abstract

Mounting evidence suggests that immune cell infiltration within the tumor microenvironment (TME) is a crucial regulator of carcinogenesis and therapeutic efficacy in ovarian cancer (OC). In this study, 593 OC patients from TCGA were divided into high and low score groups based on their immune/stromal scores resulting from analysis utilizing the ESTIMATE algorithm. Differential expression analysis revealed 294 intersecting genes that influencing both the immune and stromal scores. Further Cox regression analysis identified 34 differentially expressed genes (DEGs) as prognostic-related genes. Finally, the nine-gene signature was derived from the prognostic-related genes using a Least Absolute Shrinkage and Selection Operator (LASSO) and Cox regression. This nine-gene signature could effectively distinguish the high-risk patients in the training (TCGA database) and validation (GSE17260) cohorts (all p < 0.01). A time-dependent receiver operating characteristic (ROC) analysis showed that the nine-gene signature had a reasonable predictive accuracy (AUC = 0.707, AUC =0.696) in both cohorts. In addition, this nine-gene signature is associated with immune infiltration in TME by Gene Set Variation Analysis (GSVA), and can be used to predict the survival of patients with OC.

## INTRODUCTION

Ovarian cancer (OC) is one of the highest mortality rate malignant tumors of the female reproductive system [1]. There are more than 239,000 new cases, and about 152,000 deaths worldwide from OC every year [2]. The standard treatment plan for this disease is tumor cytoreductive surgery combined with platinum-based chemotherapy [3]. In this treatment mode, more than two-thirds of patients have a total survival of less than 10 years, and the survival rate with advanced (III-IV) stage patients is less than 20% [1].

The occurrence, development, and therapeutic efficacy of OC are closely related to many factors such as disease pathological type, TNM stage, treatment timing, and endocrine level [2, 4–6]. Most recent studies revealed genetic changes are notably linked with the occurrence and the treatment efficacy of OC. For example, from the perspective of the disease occurrence, the low-grade serous, endometrioid, clear cell, and mucinous subtypes are characterized as genetically stable, showing local invasive growth; therefore the patient has a better prognosis. About 75% of OC are high-grade serous type and genetically unstable. Majority of the patients carry p53 mutation and possible BRCA1 and BRCA2 mutations. Clinical observations of this type of patients are usually accompanied by metastatic lesions and poor prognosis [7]. From the perspective of treatment efficacy, the global loss of 5-Hydroxymethylcytosine is associated with platinum drug resistance, shortened progression-free survival (PFS), and shortened overall survival (OS) in patients with high grade serous OC [8]. OC patients with high expression of Cyclin-dependent kinase 9 (CDK9) in relapsed and metastatic lesions have a worse prognosis than patients with low expression of CDK9 [9]. A number of studies in transcription and epigenetics have confirmed that the occurrence, development, and therapeutic efficacy of OC are influenced by the dynamic changes of multiple oncogenes and tumor suppressor genes [10–15].

Existing research shows that tumor cell and host cell interaction is an important factor in promoting tumor growth and disease progression [16]. Immune cells (T lymphocytes and tumor-associated macrophages), stromal cells (fibroblasts, etc.), and extracellular matrix together form a tumor microenvironment (TME) in cancer patients [17, 18]. This TME plays a role in disease progression and formation of metastatic lesions. For example, cancer-associated fibroblasts (CAFs) facilitate OC metastasis by promoting angiogenesis, lymphangiogenesis, and tumor cell invasion [19]. CAFs induce the upregulation of Lipoma-preferred partner (LPP) in microvascular endothelial cells that can lead to chemoresistance in OC [20]. Matrix Metallopeptidase 1 (MMP1) mRNA in extracellular vesicles (EVs) secreted by OC cells can induce apoptosis of peritoneal mesothelial cells, thereby destroying the peritoneal mesothelial barrier and promoting the transfer of tumor cells to the peritoneum in OC patients [21].

Large and complicated biological data has been generated with the advent of high-throughput detection technology and bioinformatics development. The Cancer Genome Atlas (TCGA) database is one of the largest cancer genome program that provides researchers with multi-omics and standardized clinical data that can be used to design basic bioinformatics research [22, 23]. The ESTIMATE algorithm can predict tumor purity by calculating the immune and stromal scores based on specific molecular biomarker expression in both immune and stromal cells [24]. Subsequently, ESTIMATE has been applied to many neoplasms, such as prostate cancer [25], glioblastoma [26], and clear cell renal cell carcinoma [27]. However, the immune/stromal scores of OC have not been investigated in detail.

In the present study, 593 OC patients were obtained from TCGA, and their immune/stromal scores were derived from ESTIMATE algorithm. The patients were divided into high and low immune/stromal score groups with the immune/stromal score median value as the cut-off value. Differential expression analysis revealed 294 intersecting genes that influencing both the immune and stromal scores. Further univariate Cox analysis narrowed the list down to 34 genes. A final prognostic nine-gene signature was derived with a Lasso-Cox regression analysis. The prognostic nine-gene signature was trained and validated on the TCGA and Gene Expression Omnibus (GEO) datasets respectively. Time-dependent receiver operating characteristic (ROC) analysis was used to evaluate the performance of the nine-gene signature. Functional enrichment analysis and GSVA as well as Tumor Immune Estimation Resource (TIMER) were used to elucidate the valuable gene-related functions in the TME. The findings indicate that the prognostic nine-gene signature could be used as a predictive tool to assess the survival rate of patients with OC and provide novel strategies for future immunotherapy.

## RESULTS

### Clinical characteristics of the study patients

**Figure 1:**
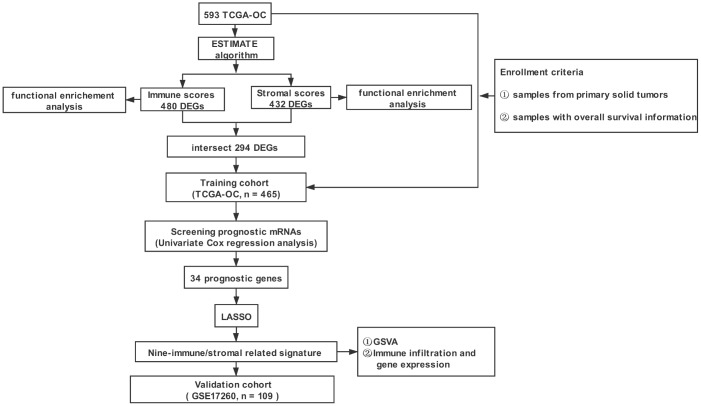
**The overall design of the study.** TCGA-OC: TCGA-ovarian serous adenocarcinoma; ESTIMATE: Estimation of STromal and Immune cells in Malignant Tumor tissues using Expression data; LASSO: least absolute shrinkage and selection operator; GSVA: Gene Set Variation Analysis.

Figure 1 shows the workflow for the identification, validation, and functional analysis of the prognostic nine-gene signature. Four hundred sixty-five OC patients from the TCGA database were included as training cohort. One hundred and nine OC patients from the GEO dataset GSE17260 were used as a validation cohort. The detailed clinical characteristics of the training and validation cohort were summarized in Table 1.

**Table 1:** Baseline characteristics of study patients.

**Variables**	**Training cohort No. (%)**	**Validation cohort No. (%)**
**No. of patients**	465	109
**Age (years)**	59.68±11.49 (mean ± SD)	
**Vital status**		
Alive	207(44.5%)	63 (58.2%)
Dead	258(55.5%)	46 (41.8%)
**FIGO stage**		
Stage II	24(5.2%)	
Stage III	362(77.8%)	92(84.5%)
Stage IV	17(16.1%)	17(15.5%)
Unknown	4(0.9%)	
**Grade**		
GB	1(0.2%)	
G1		26(23.6%)
G2	56(12.0%)	40(37.3%)
G3	397(85.4%)	43(39.1%)
G4	1(0.2%)	
Unknown	10(2.2%)	
**Venous invasion**		
NO	52(11.2%)	
YES	70(15.1%)	
Unknown	343(73.8%)	
**Lymphatic invasion**		
NO	60(12.9%)	
YES	112(24.1%)	
Unknown	293(63.0%)	
**Tumor residual disease**		
No Macroscopic disease	88(18.9%)	
1-10 mm	214(46.0%)	
11-20 mm	28(6.0%)	
>20 mm	85(18.3%)	
Unknown	50(10.8%)	

### Analysis of differential gene expression profile with immune and stromal scores in OC

**Figure 2:**
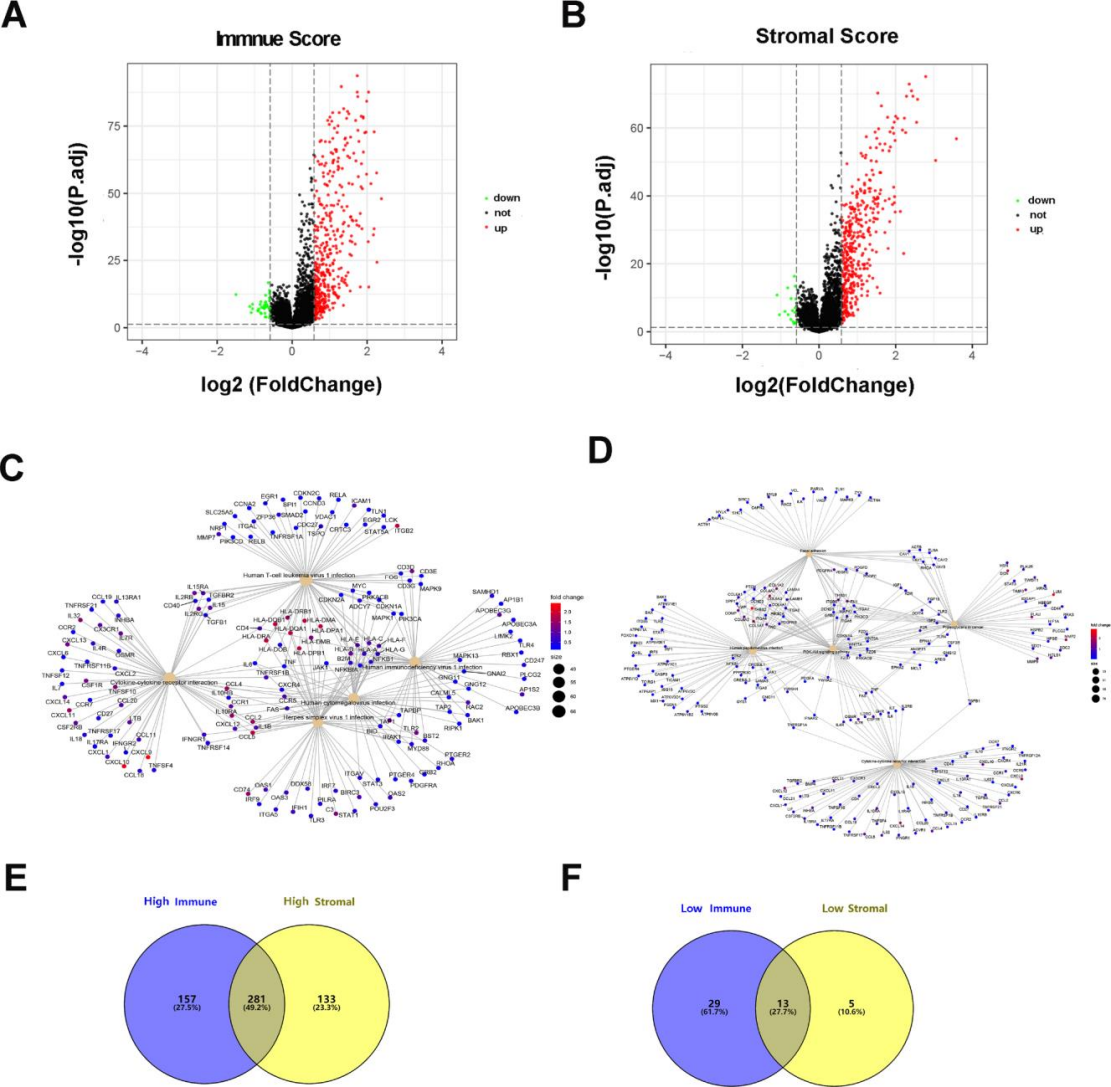
**Differentially expressed genes based on immune scores and stromal scores.** (**A**) The volcano plot showed that 438 genes were up-regulated and 42 genes down-regulated in the high immune scores group compared with the low scores group. (**B**) In a similar way, 414 upregulated genes and 18 downregulated genes were identified by comparing stromal scores. (**C**, **D**) Significantly enriched gene sets of the immune or stromal score group. (**E**, **F**) A total of 281 DEGs were in common among the high immune/stromal score groups and 13 DEGs in low immune/stromal score groups.

By comparing the gene expression profiles of patients with high immune scores against those with low immune scores, a total of 480 (438 upregulated, 42 downregulated) DEGs were identified (Figure 2A). Four hundred thirty-two (414 upregulated, 18 downregulated) DEGs were identified by comparing the high and low stromal score groups (Figure 2B). A fold-change > 1.5 and normalized p values < 0.05 were used as criterions for screening DEGs. A total of 281 DEGs were in common among the high immune/stromal score groups. A total of 13 DEGs were in common among the low immune/stromal score groups. (Figure 2E and 2F).

### DEGs functional enrichment analysis

To dissect the underlying biological function of DEGs, we performed a functional enrichment analysis utilizing the R package clusterProfiler. Immune- related 480 DEGs were significantly enriched in Human T-cell leukemiavirus 1 infection, Human immunodeficiency virus 1, Human cytomegalovirus, Herpes simplex virus 1, and cytokine-cytokine receptor interaction (Figure 2C). The statistically significant pathways of 432 stromal-related DEGs are as follows: focal adhesion, human papillomavirus infection, PI3K-Akt signaling pathway, proteoglycans in cancer, and the cytokine-cytokine receptor interaction (Figure 2D).

### Derivation of prognostic DEGs and construction of a gene risk score model

In the process of screening for prognostic-related biomarkers, the 294 DEGs in common among the high immune/stromal score and low immune/stromal score groups were subjected to a univariate Cox proportional hazard regression analysis. Out of which, 34 DEGs were found to be significantly (p < 0.05) correlated with the OS of the 465 OC patients from the TCGA database. Subsequently, these 34 candidate markers were used to construct a prognostic model with a Lasso-Cox proportional hazards regression. The resulting optimal prognostic signature for predicting the OS consists of nine genes: Ubiquitin D (UBD), V-Set And Immunoglobulin Domain Containing 4 (VSIG4), C-X-C Motif Chemokine Ligand 11 (CXCL11), Guanylate Binding Protein 2 (GBP2), C-X-C Motif Chemokine Ligand 13 (CXCL13), C-X3-C Motif Chemokine Receptor 1 (CX3CR1), Complement C5a Receptor 1 (C5AR1), Tissue Factor Pathway Inhibitor 2 (TFPI2), and DNA segment on chromosome 4 (unique) 234 expressed sequence (D4S234E). The Cox proportional hazard assumption was examined and validated through a Schoenfeld residuals test (P = 0.2259). The detailed information regarding the nine genes is provided in Table 2. The following is the formula for calculating the prognosis risk score:

**Table 2:** Nine prognostic genes significantly associated with OS in the training cohort.

**Name**	**Coefficient**	**Type**	**Down/up-regulated**	**HR**	**95%CI**	**P value**
**UBD**	-0.033	Protective	Up	0.90	0.85 - 0.96	<0.001
**VSIG4**	0.066	Risky	Up	1.14	1.05 - 1.25	0.002
**CXCL11**	-0.049	Protective	Up	0.86	0.80 - 0.93	<0.001
**GBP2**	-0.035	Protective	Up	0.90	0.82 – 1.00	0.048
**CXCL13**	-0.003	Protective	Up	0.81	0.73 - 0.91	<0.001
**CX3CR1**	0.009	Risky	Up	1.15	1.06 - 1.25	<0.001
**C5AR1**	0.009	Risky	Up	1.21	1.04 - 1.40	0.012
**TFPI2**	0.032	Risky	Up	1.08	1.01 - 1.15	0.028
**D4S234E**	-0.050	Protective	Down	0.90	0.83 - 0.98	0.011

Abbreviations: OS, overall survival; CI, confidence interval.

**Figure 3:**
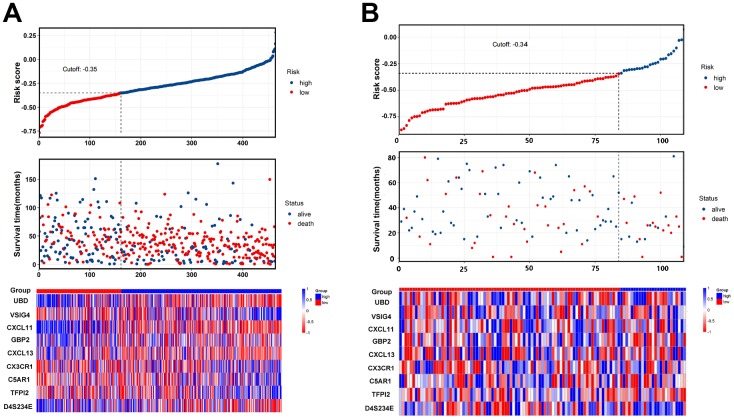
**The nine-gene signature predicts overall survival with ovarian cancer.** (**A**, **B**) The distribution of risk score, overall survival, vital status, and the heat map of the nine gene expression profile in the training cohort and validation cohort.

**Figure 4:**
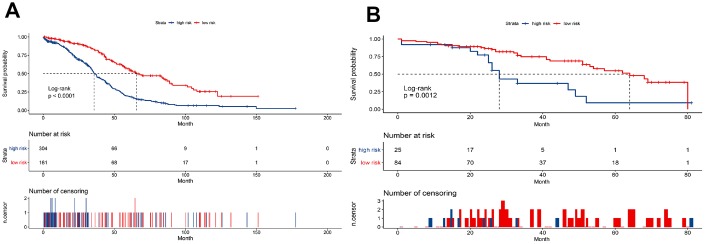
Kaplan-Meier curves to compare overall survival of high-risk and low-risk groups based on the nine-gene signature in the training cohort (**A**) and validation cohort (**B**).

Risk score = (-0.033 × expressionUBD) + (0.066 × expressionVSIG4) + (-0.049 × expressionCXCL11) + (-0.035 × expressionGBP2) + (-0.003 × expressionCXCL13) + (0.009 × expressionCX3CR1) + (0.009 × expressionC5AR1) + (0.032 × expressionTFPI2) + (-0.050×expressionD4S234E). Each patient was assigned a risk score based on the formula and divided into either high-risk group or low-risk group according to the best cut-off in two cohorts. The distribution of the gene-based risk scores, OS, OS status, and the nine-gene expression profile of the patients in the training and validation cohorts are presented in Figure 3. The heat map showed that the five protective genes (UBD, CXCL11, GBP2, CXCL13, and D4S234E) exhibit low expression in the high-risk group. In contrast, the four risk genes (VSIG4, CX3CR1, CA5R1, and TFP12) have high expression in the high-risk group. Moreover, Kaplan-Meier curves were used to compare the OS of the two groups and the analysis showed that the OS of the high-risk group was substantially shorter than the low-risk group (p < 0.001; Figure 4).

The results of the univariate and multivariate Cox proportional hazard regression analyses identified that the nine-gene signature, age, and tumor residual disease as independent prognostic variables for the OS (Table 3).

**Table 3:** Univariate and multivariate Cox proportional hazards regression analyses in the training cohort.

**Variables**	**Univariate analysis**		**Multivariate analysis**	
	**Hazard ratios (95%CI)**	***P*-value**	**Hazard ratios (95%CI)**	***P*-value**
**Age**	1.022(1.012-1.033)	**<0.001**	1.017(1.006-1.028)	**<0.001**
**Grade**				
G2	Referent			
G3	1.165(0.820-1.654)	0.394		
Unknown	1.194(0.531-2.684)	0.669		
**FIGO stage**				
II	Referent			
III	2.355(1.109-5.001)	**0.026**		
IV	2.961(1.350-6.495)	**0.007**		
Unknown	3.923(0.814-18.91)	0.089		
**Venous invasion**				
No	Referent			
Yes	0.967(0.560-1.671)	0.905		
Unknown	1.249 (0.867-1.934)	0.318		
**Lymphatic invasion**				
No	Referent			
Yes	1.264(0.798-2.001)	0.127		
Unknown	1.094 (0.732-1.636)	0.374		
**Tumor residual disease**				
No Macroscopic disease	Referent		Referent	
1-10 mm	1.899(1.324-2.722)	**<0.001**	1.469(1.006-2.144)	**0.046**
11-20 mm	2.191(1.259-3.814)	**<0.001**	2.034(1.140-3.629)	**0.016**
>20 mm	2.313(1.536-3.483)	**<0.001**	1.803(1.177-2.762)	**0.007**
Unknown	0.975(0.595-1.597)	0.919	1.034(0.626-1.709)	0.896
**Nine-mRNA signature**	21.48(10.15-45.42)	**<0.001**	15.60(6.963-34.96)	**<0.001**

### Risk score model accuracy assessment

**Figure 5:**
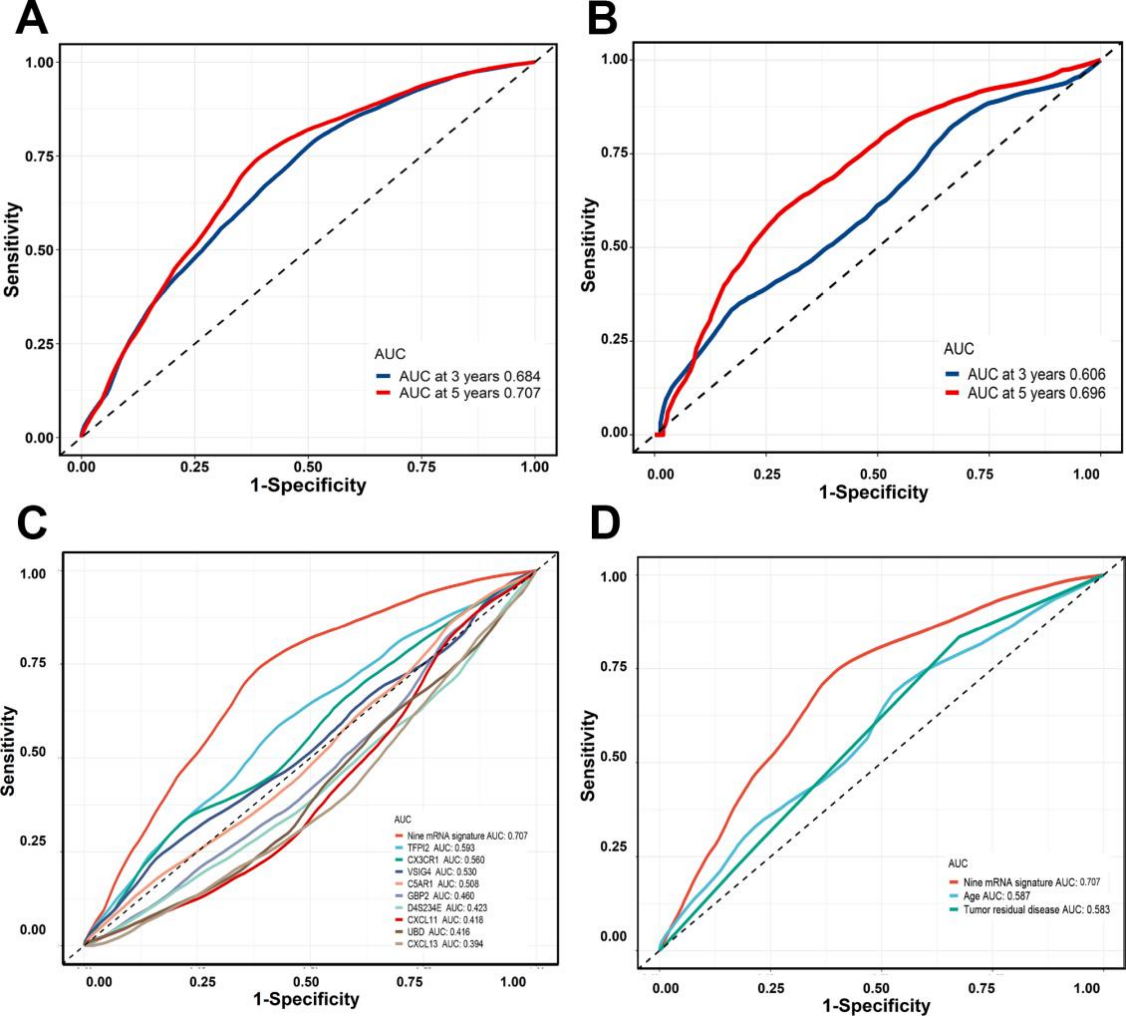
**Time-dependent ROC curves were generated to evaluate the nine-gene signature performance.** (**A**, **B**) Three-years or five-years ROC curves of the nine-gene signature in the training cohort and validation cohort. © Five-years ROC curves for nine-gene signature and single gene. (**D**) Five-years ROC curves for nine-gene signature and clinical risk factor.

The time-dependent ROC curve analysis was conducted and the area under the curve (AUC) value was used to evaluate the predictive effect of the nine-gene signature. In the training cohort, the three-year AUC was 0.684 and the five-year AUC was 0.707. In the validation cohort, the three-year AUC was 0.606 and the five-year AUC was 0.696 (Figure 5A and 5B). By comparing the nine-gene signature against other prognostic factors and single gene individually, the nine-gene signature demonstrated a higher prognostic accuracy (Figure 5C and 5D).

### Comparing the immune infiltration between the high- and low-risk groups

**Figure 6:**
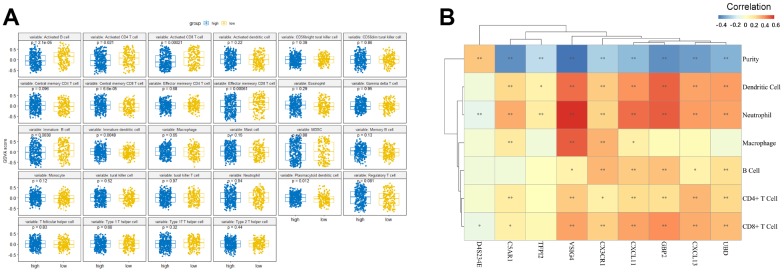
**The relationship between the nine-gene signature and immune infiltration.** (**A**) Comparison of relative immune cell abundance based on GSVA score in high-risk and low-risk groups (**B**) Partial Spearman's correlation of nine genes expression and immune infiltrates. *: Statistically significant p < 0.05, **: Statistically significant p < 0.01.

To provide novel insight into the biological role of each of the risk groups, we performed immune infiltration analysis using the GSVA method. Among the low-risk group, the level of immune infiltration (e.g., “Activated B cell”, “Activated CD4 cell”, “Activated CD8 cell”, “Effector memory CD8 T cell”, and “Immature B cell”) were found to be significantly higher than that of the high-risk group (Figure 6A). In contrast, “Central memory CD8 T cells”, “Immature dendritic cells”, and “Plasmacytoid dendritic cells” were significantly enriched in the high-risk group (Figure 6A).

Since tumor-infiltrating lymphocytes are an independent prognostic predictor of survival in various tumors [28–30], we performed correlation analysis between the nine genes and immune infiltration level for OC. The results showed that the association between the nine genes and the immune microenvironment is significant, as each of the nine genes had a significant correlation with tumor purity (Figure 6B). Among these genes, D4S234E was positively correlated with tumor purity, whereas the other eight genes were negatively correlated with tumor purity. The most relevant genes among the nine gene signature associated with immune infiltration included: CX3CR1 (related B cell, cor = 0.311), GBP2 (related CD8^+^ T cell, dendritic cell, cor = 0.403, 0.495), CXCL13 (related CD4+ T cell, cor = 0.308), and VSIG4 (related macrophage and neutrophil cor = 0.51, 0.605).

### Stratification analysis based on clinical information

Risk stratification analysis was performed to test whether the nine-gene signature could predict OS regardless of tumor residual disease. The results shown that patients with low-risk scores had significantly longer OS than patients with high-risk scores in no macroscopic disease (p=0.0014, 1-10 mm (p =0.00026), >20 mm (p=0.0001)) (Supplementary Figure 1).

## DISCUSSION

OC represents one of the diseases with the highest mortality rate of the female reproductive system [1]. Due to the lack of early and effective detection methods, most OC patients were diagnosed at an advanced stage who subsequently missed the optimal treatment period and resulting in a poor clinical outcome. Recent studies have shown that TME played a vital role during OC progression [31–33]. Moreover, TME-related molecular markers can be used as predictors to precisely assess patients’ immunotherapy response, thereby enhancing their clinical outcome [34–36]. However, immune infiltration and its molecular mechanisms have not been thoroughly explained in OC.

To our knowledge, our work is the first to use the ESTIMATE algorithm combined with LASSO-Cox to explore molecular markers associated with OC prognosis. Firstly, we derived a series of TME-associated DEGs by comparing the transcriptional expression profiles in 593 OC patients with high versus low stromal/immune scores based on TCGA data. The DEGs functional enrichment analysis indicated that the main pathways were associated with immune response and cancer (e.g., cytokine-cytokine receptor interaction, Human immunodeficiency virus 1 infection, focal adhesion, proteoglycans in cancer, and PI3K-Akt signaling pathway), which are in agreement with findings that immune response and cancer progression exhibit crosstalk and interact with each other [37]. Based on the obtained DEGs, we built a nine-gene signature that was notably related to the OS in OC patients in both the training and validation cohorts. The patients could be divided into high-risk and low-risk groups with distinct differences in the five-year OS with this nine-gene signature. The results of the GSVA analysis showed that “Activated CD8 T cells”, “Effector memory CD8 T cell”, “Activated B cells”, and “Activated CD4 T cells” were associated with significantly lower infiltration in the high-risk group. In contrast, “Central memory CD8 T cells”, “Immature dendritic cells”, and “Plasmacytoid dendritic cells” were associated with significantly higher infiltration in the high-risk group.

Numerous studies have documented that CD8^+^ T cell high infiltration in the TME is associated with positive anti-tumor effects in various cancer [38–40]. Natural killer (NK)-dendritic cell (DC) cross talk results in upregulation of Chemokine (C-X-C motif) ligand 9 (CXCL9), Chemokine (C-X-C motif) ligand 10 (CXCL10), and Chemokine (C-C motif) ligand 5 (CCL5) on DCs leading to CD8^+^ effector T cells recruitment into TME, thereby promote antitumor immune response in OC [41]. On the other hand, the expression of inhibitory molecules such as CTLA4, PD-1, and LAG3 on CD8^+^ T cells are promoted by IL-6 and IL-10, that produced by tumor cells and tumor-associated macrophages, in turn inhibit CD8^+^ T cells infiltration [42–44]. Another example of CD8^+^ T cells inhibition shown C-C motif chemokine 22 (CCL22) can promote CTLA4^+^ FOXP3^+^ GITR^+^ Tregs and CCR4+ Tregs infiltration in TME, thereby inhibiting CD8^+^ T cells activation [45, 46].

Curdin et al. reported that plasmacytoid DCs can induce immunosuppression in OC by providing ICOS^+^ Treg cells with Inducible T Cell Costimulator Ligand (ICOS-L) stimulation, thereby enhancing the capability of Treg cell impairments of T cell proliferation [47]. Immature DCs express low levels of Major histocompatibility complex (MHC) and co-stimulatory molecules, therefore T cells activation by immature DCs is inefficient [48, 49]. These results suggest that a low density of activated T cells or high infiltration of immature/plasmacytoid DCs may be the cause of poor clinical outcome of cancer patients.

Tumor progression and metastasis typically occur in adipose tissue-rich areas such as omentum, one of the main metastatic sites in OC. Adipose tissue is composed of a variety of cells including adipocytes, adipose stem cells, endothelial cells, and infiltrating immune cells that secrete diverse soluble tumor-promoting factors such as hormones, cytokines, reactive oxygen species, extracellular matrix, and lipid metabolites. These secreted factors not only directly promote tumor progression but can also reduce the anti-tumor immune response by altering the TME. For example, chemokines (e.g., TNF-α, IL-6, and IL-1b) recruit immunosuppressive neutrophils and M2 macrophages to the TME, thereby inhibiting anti-tumor cell activity (e.g., TCD 8+ lymphocytes and NKTs) [50]. Besides, adipose tissue associated PD-L1 is found to attenuate T cell activation which also contributes to an immune suppressive microenvironment [51]. A large number of adipose tissue infiltrating M1 macrophages can lead to adipocyte death. Moreover, the release of intracellular substances of dead adipocyte not only aggravates inflammation but also provides energy required for tumor cell growth. These factors all provide a favorable microenvironment for tumor growth [52].

Cancer is a heterogeneous disease for which the identification of dysregulated genes involved in tumorigenesis and progression might aid in improving prognostic and treatment strategies. In this study, we identified a group of nine genes (CX3CR1, UBD, GBP2, D4S234E, CXCL11, CXCL13, VSIG4, TFPI2 and C5AR1) that can effectively predict the OS in OC. Among these genes, CX3CR1 and UBD can promote tumor metastasis and the epithelial to mesenchymal transition [53–55]. As p53-related genes, GBP2 and D4S234E have been previously shown to regulate mitochondrial fission and apoptosis of cancer cells [56–58]. CXCL11 and CXCL13 are associated with CD8^+^ T cell and B cell infiltration, which act as a protective factor inhibiting tumor [59, 60]. VSIG4 inhibits T cell proliferation and IL-2 production as well as regulates Treg differentiation and stability leading to immune tolerance [61, 62]. TFPI2 is a serum biomarker for the detection of ovarian clear cell adenocarcinoma, and its predictive values are superior to that of CA125 [63]. Great promise for immunotherapies has been achieved by the Conduct Phase II and III clinical trials for the discovery of a drug that targets the C5a-C5aR1 pathway [64]. Therefore, our nine-gene signature could potentially be used as a predictive tool for risk assessment and might offer potential targets for immunotherapy in the clinical management of OC.

There are also some limitations associated with this research that should be addressed. First, the biological function of the nine identified genes should be validated in wet lab experiments, particularly regarding the association with immune infiltration. Second, missing information in OC clinical characteristics (contains many patients with “unknown” information in venous invasion and lymphatic invasion) in TCGA limited us in building a nomogram for incorporating clinical characteristics to improve the predicted accuracy of the model. Third, the risk score model requires further validation in multiple cohorts to evaluate the model generalization ability.

In conclusion, the gene expression profile and clinical characteristics of the TCGA database were analyzed by ESTIMATE and a Lasso-Cox algorithm to obtain the nine gene prognostic signature related to TME in OC. This molecular signature can effectively distinguish high-risk populations from OC patients in TCGA and GSE17260 datasets. In addition, the expression of each gene in the model is significantly correlated with the TME components, which further supports the important role of the TME in the occurrence and development of OC.

## MATERIALS AND METHODS

### Data source

The available Level 3 gene expression profiles of the OC patients were downloaded from the TCGA database (https://tcga-data.nci.nih.gov/tcga/). RNA expression detection of ovarian serous adenocarcinoma was performed on Affymetrix HT Human Genome U133a microarray. Clinicopathological characteristics including age, histological type, FIGO stage, venous invasion, lymphatic invasion, tumor residual disease, survival, and outcome were also downloaded from the TCGA data coordination center. ESTIMATE algorithm was used to calculate immune and stromal scores using "estimate" package (http://r-forge.rproject.org; repos= rforge, dependencies=TRUE) [24].

There were 593 ovarian cancer samples with gene expression data in the TCGA. The data from 576 primary solid tumors patients were retained after removing 17 recurrent solid tumors samples. The samples were further trimmed to 465 to include only patients with corresponding clinical information. The distribution of the values for the samples we have selected was viewed graphically as a box plot and no outliers were identified. All 465 serous adenocarcinoma samples contained complete survival information (survival time and outcome). All 12,042 coding genes of each patient did not contain missing values. Gene expression values were normalized by log2 (Affy RMA). The 465 OC patients were grouped as a training cohort. To confirm whether our nine-gene signature could predict prognosis of OC patients in an independent dataset, we selected to use publicly available data (GSE17260) with clinical informations such as OS time, samples were diagnosed as serous adenocarcinoma, obtained from primary lesion, and gene expression profiling was measured by microarray. The raw data for the validation set GSE17260 was downloaded from the GEO database, which include 109 OC samples (a low quality sample was removed) and its corresponding clinical information (Grade, FIGO stage). R package limma was used for quality control and normalization. Gene expression value for genes with multiple probes was calculated as the average of the probes.

### Identification of prognosis-related genes

We divided the patients into two groups based on the median value of the immune scores and stromal scores. The differential expression analysis was performed with R package limma [65], and the fold-change (> 1.5) and an eBayes test p-value (< 0.05) were used as criteria to screen for DEGs between the high and low groups. We subsequently performed a functional enrichment analysis with the R package “clusterprofiler” [66] to identify the potential biological function of the DEGs. The intersection of immune and stromal DEGs was subjected to a univariate Cox regression analysis to identify the OS-related signature. A threshold of p < 0.05 was deemed significant.

### Construction of risk score system of OC

We performed the least absolute shrinkage and selection operator (LASSO) on the Cox regression model using R package glmnet [67]. The 10-fold cross-validation approach and “one-standard error (1se)” was used to identify the optimum parameter λ. After the Lasso-Cox analysis, we obtained the corresponding regression coefficients and signatures of the nine genes and constructed the following formula:

Risk score = sum of each gene^’^s (regression coefficients × level of gene expression)

Each patient was assigned a risk score according to this formula. The optimal cut-off of the risk score was determined by the “surv_cutpoint” function of the “survminer” R package (https://www.r-project.org/) and used to stratify the OC patients into high- and low-risk groups. A comparison of the survival between the two groups was analyzed with a Kaplan-Meier estimator and log-rank test.

### Time-dependent ROC curve analysis

A time-dependent ROC Curve method can be implemented to estimate the three- and five-years prognostic model prediction performance in a training and validation cohort [68]. A stratified analysis was conducted to investigate whether the prognostic model was widely applicable to clinical characteristics. Furthermore, the AUC was used to determine whether the prognostic model was superior to that of other risk factors.

### The correlation between gene expression and immune infiltration

Gene sets for 28 subpopulations of tumor-infiltrating lymphocytes resulting from the study by Charoentong et al. [69], containing cell types related to adaptive immunity (activated, central memory, effector memory CD4^+^ and CD8^+^ T cells, γδ T cells, type 1 helper T (TH1) cells, TH2 cells, TH17 cells, regulatory T cells, follicular helper T cells, as well as activated immature and memory B cells) and innate immunity (macrophages; monocytes; mast cells; eosinophils; neutrophils; activated, plasmacytoid and immature dendritic cells; natural killer cells; natural killer T cells, and myeloid-derived suppressor cells). The gene set parameter was subjected to GSVA analysis. GSVA transformed gene expression into an absolute enrichment score, which was represented as relative immune cell abundance in each sample [70]. A t-test was performed to compare the GSVA score between the high- and low-risk groups. The Tumor Immune Estimation Resource (TIMER, https://cistrome.shinyapps.io/timer/) was used to investigate the correlation between gene expression and tumor-infiltrating immune cells, including B cells, CD4^+^ T cells, CD8^+^ T cells, neutrophils, macrophages, and dendritic cells in OC [71]. A heat map was generated with partial Spearman's correlation and p < 0.05 was regarded as statistically significant.

## Supplementary Material

Supplementary Figure 1
