# Supplementary material for: A nine-gene signature related to tumor microenvironment predicts overall survival with ovarian cancer
Source: Aging (Albany NY). 2020 Mar 24;12(6):4879–95. doi: 10.18632/aging.102914 (PMC7138578; doi:10.18632/aging.102914)
Supplement: Supplementary Figure 1 [file aging-12-102914-s001..pdf]

SUPPLEMENTARY FIGURE

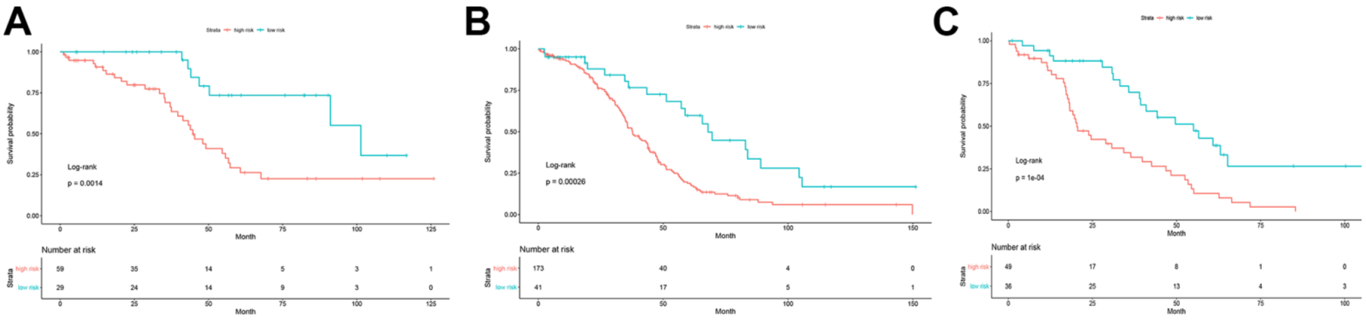

Supplementary Figure 1. Kaplan-Meier curves of OS in the subgroups stratified by tumor residual disease. (**A**) No Macroscopic disease (**B**) Tumor residual size is 1-10 mm (**C**) tumor residual size >20 mm.
